# Correction: Recruitment of the default mode network during a demanding act of executive control

**DOI:** 10.7554/eLife.29017

**Published:** 2017-06-05

**Authors:** Ben M Crittenden, Daniel J Mitchell, John Duncan

Crittenden BM, Mitchell DJ, Duncan J. 2015. Recruitment of the default mode network during a demanding act of executive control. *eLife* **4**:e06481. doi: 10.7554/eLife.06481.

Published 13, April 2015

Recently a reader noticed a discrepancy between our described method of data normalisation prior to pattern classification and the default method of the software toolbox (the decoding toolbox) which we used. We did, in fact, use the default method, but due to a misunderstanding of the code, reported the normalisation incorrectly. Specifically, in the last paragraph of the methods section, we described the normalization as: ‘Prior to pattern analysis within each ROI, beta values were Z-scored across all voxels within the ROI, separately for each task’. This should have read: ‘Prior to pattern analysis, beta values were Z-scored across tasks within each voxel of the ROI’. The results remain unchanged.

The article has been corrected accordingly.

